# Integration of Traditional Birth Attendants into Mental Healthcare: A Multistakeholder Qualitative Study Exploration

**DOI:** 10.1155/2019/8195267

**Published:** 2019-03-21

**Authors:** Christine W. Musyimi, Victoria N. Mutiso, Darius N. Nyamai, Ikenna D. Ebuenyi, David M. Ndetei

**Affiliations:** ^1^Africa Mental Health Research and Training Foundation, Nairobi, Kenya; ^2^Vrije Universiteit Amsterdam, Amsterdam, Netherlands; ^3^University of Nairobi, Nairobi, Kenya

## Abstract

**Background:**

A significant number of people with common mental disorders are undiagnosed or undetected at primary healthcare facilities. The experience of traditional birth attendants (TBAs) in reassuring perinatal mothers could be utilized in maternal mental healthcare. The aim of this study was to gain insight into the feasibility of integrating TBAs into maternal mental healthcare using multiple stakeholder views.

**Methods:**

We conducted an exploratory qualitative study in September 2017 using focus group discussions (FGDs) and in depth interviews in Makueni County, Kenya. A total of 246 participants (TBAs, community health volunteers (CHVs), healthcare workers (HCWs), antenatal and postnatal mothers seeking care from TBAs and those seeking both hospital and TBA services, mothers in law and/or husbands of perinatal mothers, and opinion leaders based in the county) were purposively selected to participate in the discussions. Transcribed data was analyzed using NVivo version 10.

**Results:**

Four major themes emerged from the qualitative data and were identified as follows; (a) involving TBAs in perinatal mental healthcare by assigning them roles, (b) utilizing TBAs' patient rapport and counseling experience, (c) recognition and appreciation of TBAs by the healthcare system, and (d) training and collaboration of TBAs with healthcare workers.

**Discussion:**

The findings of this study reveal that although TBAs informally provide psychosocial interventions to pregnant mothers, their roles in mental health are not clearly defined. The importance of TBAs sharing their experience and being recognized as important stakeholders in mental healthcare for perinatal mothers was highlighted. Inclusion of TBAs in dialogue and training them to offer evidence-based mental healthcare were identified as important steps towards improving the mental wellbeing of mothers and the future generation.

## 1. Introduction

It is evident that global disease burden is changing due to the shift in disease occurrence pattern. Mental, neurological, and substance use (MNS) disorders are claiming a significant share with associated disability [1]. In 2010, MNS accounted for 10.4% of disability-adjusted life years (DALYs) as the highest cause of years lived with disability (YLDs) [2]. One of the MNS disorders is depression, classified by World Health Organization (WHO) as a priority MNS condition for intervention, based on the evidence of the disease burden in terms of morbidity, disability, and the resulting negative economic consequences [3]. Its prevalence during pregnancy is associated with factors like unwanted pregnancies, poverty, lack of support from the family during prenatal period [4] and conflict with the partner [5]. Moreover, previous history of depression is associated with increased risk of perinatal depression [6], more so among adolescents [5]. Fisher and colleagues established that women living in low middle income countries (LMICs) are more susceptible to the nonpsychotic common perinatal mental disorders (CPMDs), with a prevalence of 15.6% in antenatal and 19.8% in postnatal mothers [7]. A systematic review involving eight African countries also indicated that depression is the commonest assessed perinatal mental health disorder with a prevalence of 11.3% and 18.3% among antenatal and postnatal mothers respectively [8]. Evidence also points to an elevated incidence of depression in the first few weeks postnatally [9].

In Kenya, the prevalence of common mental disorders is on the rise with estimates of nearly 25% for depression in community settings [10] and 42% in healthcare settings [11]. Unfortunately, universal mental healthcare is hindered by scarcity of resources. This is portrayed in the documented trend of inadequate funding for mental health programs and human resources, which is a common feature in most LMICs [2]. In this regard, the scenario in Kenya indicates a dearth of mental healthcare personnel that is unable to match the relatively high psychiatric morbidity [12]. A significant number of people with MNS are undiagnosed or undetected at the general healthcare facilities due to infrastructural constraints [11] with maternal mental health problems affecting a significant number of women, hence posing severe emotional, physical, and economic consequences on the families and affecting the children's behavior and psychological development. Infants of mothers who suffer from maternal depression have poor mental development and growth rate, higher rates of diarrheal diseases, and other childhood illnesses [13, 14]. Mothers with depression are also more likely to stop breastfeeding earlier than the recommended duration [15]. In a far reaching consequence, maternal depression is associated with a higher risk of depression for the spouse and contributes to strained social and emotional family atmosphere [16]. It is therefore a matter of concern to fortify the identification and treatment of depression appropriately and especially during the perinatal period.

In view of the prevalence of depression contrasted by the inadequate care and threat to maternal child wellbeing, bridging the treatment gap should be highly advocated. The feasibility of involving the informal health providers (IHP) by building their capacity to provide psychosocial intervention for patients with depression has been ascertained [17]. Through task sharing models, mental healthcare can be provided by nonspecialists and this would be helpful in bridging the treatment gap that exists especially in under-resourced settings [18]. Setting up these effective intervention models and treatment coverage calls for innovative approaches and collaboration with all key community healthcare providers, including traditional and religious groups [19]. It is encouraging that nonspecialist healthcare workers and community healthcare providers can be effectively utilized in providing the much needed evidence-based intervention for maternal depression [20].

In Kenya, there has been a growing shift away from engaging traditional birth attendants (TBAs) in maternal healthcare particularly conducting deliveries but encouraging hospital referrals. Nevertheless, pregnant mothers still enjoy a cordial relationship with TBAs due to the personalized care and rapport that makes the TBAs approachable. This group of IHP has been dominated by elderly women who are held in high regard by the society they live in. Undoubtedly, their experience which is gained during years of practice could be utilized in maternal healthcare, especially in poor-resourced rural settings [21]. In spite of government policy encouraging hospital delivery by skilled birth attendants, TBAs continue to receive perinatal mothers who visit for counseling, massaging, and emotional support [22]. Earlier literature has revealed that TBAs have provided interventions informally in terms of reassuring and counseling their clients who present with perinatal stress symptoms. However, there is paucity of research related to the community views on integrating them into mental healthcare. This study focused on generating qualitative evidence pertaining to the community views and feasibility of integrating TBAs into maternal mental healthcare.

## 2. Materials and Methods

### 2.1. Study Design, Population, and Site

We conducted an exploratory qualitative study in September 2017 using focus group discussions (FGDs) and in depth interviews in Makueni County, a rural setting located on the lower Eastern part of Kenya. Makueni is one of the 47 counties in Kenya with a population of about one million and is divided into six administrative areas referred to as subcounties.

### 2.2. Recruitment

In consultation with county department of health, two out of the six Makueni subcounties were selected based on nonexistence of formal mental health activities. Participants were purposively selected in the two subcounties by the subcounty public health officer and included traditional birth attendants (TBAs), community health volunteers (CHVs) commonly known as community health workers, healthcare workers (HCWs) located in busy health facilities in the two subcounties, antenatal and postnatal mothers seeking care from TBAs and those seeking both hospital and TBA services, mothers in law and/or husbands of perinatal mothers, and opinion leaders based in the county.

Selection of TBAs was based on being proactive in community activities such as referral of mothers to hospital for delivery and regular attendance of monthly meetings with the public health officer. Subsequently, the selected TBAs identified and selected antenatal and postnatal mothers from the list of mothers who had visited them. In turn, the antenatal and postnatal mothers invited their caregivers (mothers in law or husbands) for participation in discussions. The discussions which took between 45 to 90 minutes explored the experiences of women regarding use of TBA mental health services and perspectives of the key stakeholders regarding the integration of TBAs into mental healthcare, while giving room for probing, to gain an in depth understanding and clarity on the topic. The audio-recorded discussions were transcribed and translated by a multilinguist fluent in spoken and written local language* (Kikamba).* Saturation of theme [23] was achieved after conducting at least three FGDs and atmost 6 FGDs with different categories that included traditional birth attendants (TBAs), community health volunteers (CHVs), healthcare workers (HCWs), pregnant women, postnatal women, and caregivers (husbands, mothers, and mothers in law) (Table 1). A recent study by Guest and colleagues also found out that 90% saturation of themes is achieved within three to six FGDs for each category of participants [24].

Ethical approval was sought from Maseno University Ethics Review Committee (MUERC) while written consent was obtained from all participants.

### 2.3. Data Analysis

Two authors read through all the transcripts and independently coded the data by use of NVivo version 10. Thematic content analysis was utilized and different themes were identified from the data which was shared with the rest of the authors. The final four themes agreed upon by all the authors are discussed in the results section.

## 3. Results

The study participants responded to the research questions such as: In your opinion, what role do TBAs play in the community? What is your opinion on integrating TBAs into mental healthcare? How can you describe the relationship between TBAs and the ministry of health? What collaborative initiatives between TBAs and the ministry of health would you consider?

Four major themes emerged from the analysis:

- involving TBAs in perinatal mental healthcare by assigning them roles
- utilizing TBAs' patient rapport and counseling experience
- recognition and appreciation of TBAs by the healthcare system
- training and collaboration of TBAs with healthcare workers

*(a) Involving TBAs in Perinatal Mental Healthcare by Assigning Them Roles. *Responding to the question about the TBAs' role in the community, stakeholders identified the absence of a clear job description for the TBAs and lack of structured division of responsibilities. Participants were cognizant of the existence of TBAs and indicated the need for involving them in healthcare system by defining their role. This perception was captured by a 56-year-old mother in law of a pregnant mother during a FGD:

“Before the healthcare workers were in existence, a TBA was still there… They should sit down (with informal healthcare providers) and ‘solve the equation'; agree, on the specific roles for each one of them.”

A 36-year-old husband of a pregnant mother also described the lack of clear roles for TBAs despite their active engagement in community health activities:

“… I have not heard a TBA who has ever been given a role by healthcare workers, yet I see them (TBAs) escorting pregnant women to the hospital.”

Similarly, a 30-year-old clinician based at a health centre suggested: “*I think as health workers and TBAs we need to define the roles of each other.*”

If a clear working engagement between TBAs and HCWs is formulated, this collaboration could benefit the community. A direct beneficiary of healthcare providers' antenatal services aged 31 states,

“The healthcare workers should have a discussion with TBAs and agree so that they can work together for the benefit of the people in communities”

TBAs could come in handy to offer the required support especially in solving family conflicts. This is important in maintaining good mental health during perinatal period and reducing the risk of maternal depression and possibly developmental disorders in the offspring. A 57-year-old healthcare worker described how TBAs could be resourceful in dealing with stressors at the family level:

“You find that some families have problems. For instance, the mother is pregnant and when she disagrees with the husband she has nobody else to go to for support but if the TBAs are acknowledged, she can go to her and share her problems which could reduce the chances of having depression among pregnant women.”

*(b) Utilizing TBAs' Patient Rapport and Counseling Experience. *With further probing on the same question of TBA role in the community, another theme emerged which specified that TBAs have a long experience of working with the community and they understand its dynamics.

“TBAs should be given an opportunity to share their experiences as some of them are already in registered self-help groups.”

The involvement of TBAs in identification and addressing psychosocial stressors among mothers was highlighted in the discussions. This was viewed as crucial in improving the mental wellbeing of the mothers during the prenatal period. A postnatal mother aged 23 stated,

“TBAs can be involved (in mental healthcare) because sometimes when a TBA is around she can offer guidance and counseling. They counsel you and enable you to know what the problem is.”

A 55-year-old TBA observed: “*We counsel people with mental illness and those with many thoughts… we sit them down, chat with them to identify their problem. We then give them talk therapy and tell them how they can lead normal lives*.”

Another TBA, a widow aged 36 years, commented on how they deal with suicidal ideation among pregnant mothers*, “After counseling her, we go to that family and talk to those people close to her….”*

An 18-year-old adolescent who had attempted suicide commented,* “A TBA counseled me and I got courage to move on with my life.”*

*(c) Recognition and Appreciation of TBAs by the Healthcare System. *The study participants were asked to describe the relationship between the TBAs and the ministry of health and pointed out that TBAs are not accorded the recognition they deserve by the healthcare system. They identified the lack of documentation of their community involvement in health-related activities such as the referral of pregnant mothers. An elderly TBA (65 years) firmly explained,

“There are no records (at the hospital) when we refer mothers to hospitals to show how active we have been in community activities.”

The participants also stated that there are no regular meetings or forums for sharing knowledge or receiving updated information. An interview with a key informant in her mid-fifties revealed how the TBAs would feel appreciated if a recognized opportunity for having structured groups could be availed:

“We will feel appreciated by having a structured group of TBAs where we can share, initiate something jointly and perhaps receive regular updates on upcoming community activities.”

The TBAs also said that they do not have any form of identification when they take the pregnant mothers to the hospital and felt a need to be recognized during the referral process. A 62-year-old TBA states,

“It's just terrible. We are sidelined as TBAs. We need identification cards to identify us when we take the pregnant mothers to the hospital.”

Healthcare workers (as described by a female nurse in her late fifties) also acknowledged the lack of appreciation for the TBAs and suggested the need to enhance this by promptly offering assistance to TBAs whenever they escort a patient to the hospital, by viewing them as coworkers and respectfully offering culturally acceptable greetings, promptly guiding and assisting them.

“When the TBAs come to the health centre we recognize them as our coworkers, we greet them with respect. For instance, sometimes you pick her (TBA) in the queue and acknowledge her so that she feels recognized. So that kind of recognition may even be more than salaries to them because they are recognized as core workers by the health facility”

*(d) Training and Collaboration of TBAs with Healthcare Workers. *The participants were asked whether TBAs should be integrated into mental healthcare and also their opinions on collaborative initiatives between the TBAs and the ministry of health. In their response, the inclusion of TBAs in the management of perinatal depression was considered relevant on account of their proximity and the close relationship they have with the mothers with depression in their community. According to a 55-year-old female CHV,

“I would say they are supposed to be involved (in mental healthcare) because mostly they are very close to these people with depression.”

Close collaboration between TBAs and formal healthcare workers is essential. TBAs can be involved in offering first-line intervention for perinatal depression before referral to the hospitals since they are readily accessible whenever a psychosocial need arises. An antenatal mother in her early twenties explained,

“TBAs should be involved because they are very close to the communities and can always be reached for advice. Then from there the mothers can go to the hospital.”

TBAs are accepted as “grass root doctors” trusted by the community members. The community appreciates that the healthcare practitioners may not be in a position to offer the home- based interventions due to their busy schedules as opposed to the TBAs. If well trained, the TBAs could identify the patients' psychosocial stressors that contribute to symptoms at an early stage and address them before they become full-blown. In a FGD, a 37-year-old TBA described,

“If possible, a TBA should be taught how to handle these people with mental disorders. This is because a TBA is the “doctor” at the grassroots. No matter how a healthcare worker is skilled he/she cannot get time to go to the village. But a TBA can meet with the woman on a daily basis if need arises as they live in her neighborhood. This can be good because this problem will be treated very fast before it becomes full-blown.”

Cooperation between the healthcare workers and the TBAs will be to the advantage of the society and therefore, working together is in essence a crucial step to long-term collaboration. A young new mother (20 years) stated in a FGD,

“TBAs should cooperate (with HCW) so that many activities can move on smoothly.”

Training of TBAs to offer interventions for common mental disorders during the perinatal period could be helpful in reducing the work load at the strained primary healthcare facilities. A 31-year-old female nurse in a FGD enthusiastically stated,

“Including them (TBAs) in management of mental health services is important because they may help reduce workload for us as staff in the facility.”

*“(Involving TBAs in mental health issues) will help us in the follow-up of these clients at home.” *Another 24-year-old male nurse described the importance of TBAs conducting patient follow-up to relieve the overwhelmed healthcare staff.

Training of TBAs and inviting them for seminars will enable the process of monitoring their community health related activities. A 56-year-old female CHV emphasized the need for training TBAs:

“TBAs should be [taught] more skills and invited in seminars so that they are monitored.”

Training the TBAs can help to recognize the importance of team work and enable them become part of an effective healthcare system. This will make them appreciate other health related issues and foster a good relationship with other healthcare practitioners. TBAs' training on mental health and inclusivity in the service provision could also motivate and increase their zeal in the service to the community as explained by the TBAs.

“TBAs can be trained that being a TBA is not an independent unit but it's a multidisciplinary service for many other professions.” (45-year-old TBA)

This perspective was also shared by another TBA who suggested the following:* “If we can be trained on mental disorders, we can feel motivated….” (62-year-old TBA)*

The utility of TBAs in the community healthcare and the importance of training them on evidence-based mental healthcare practice were also highlighted by the caregivers of perinatal mothers.

“TBAs are useful to the society and should be trained on good mental health practices to help doctors,” stated a 75-year-old mother in law of a postnatal mother.

A well-tailored training that fits the needs of the society while taking into account the capacity and abilities of the participating TBAs especially the language can be beneficial in gaining skills in the management of mental health disorders. A 25-year-old pregnant mother emotively explained,

“Training can be offered to the TBAs. I am not saying that they are skilled but there is a way that they can be trained using their own native language to gain those skills.”

## 4. Discussion

Similar to previous research work, the findings of this study pointed out that TBAs provide various different services [22] including counseling and reassuring pregnant mothers. Despite their involvement in counseling, their contribution in mental healthcare is not recognized. The stakeholders reiterated the importance of TBAs sharing their experience and being recognized as important stakeholders in mental healthcare for perinatal mothers. The inclusion of TBAs in dialogue with healthcare workers and providing training on evidence-based mental healthcare was viewed by stakeholders as a step towards improving early diagnosis and avoiding complications.

Recent evidence reveals that treatment pathways for patients with mental disorders should be improved by taking into account the role of informal health providers in the community and building a collaborative network [19]. Our findings indicate that TBAs have been involved in offering counseling and emotional support to perinatal mothers who approach them for help. They support mothers who are undergoing distressing family issues by listening to them and providing guidance in solving the problems. This practice is reassuring and it endears them to the perinatal mothers because they are not able to receive such visits from the healthcare workers. This observation is corroborated by a similar study conducted in rural Kenya [21] and this could be an indication that TBAs have some level of experience in counseling and community problem solving techniques which can be harnessed in low-mental health resource settings.

According to the study findings, the rationale for integrating TBAs into mental healthcare was also strengthened by the fact that TBAs are easily accessible and accepted and have a good understanding of the community dynamics. These findings are similar to what Imogie and colleagues described in another study from Sub-Saharan Africa [25]. Therefore, the capacity in service delivery should be enhanced through appropriate training that takes into account local contextual issues of beliefs and practices [25]. These approaches have been successful in task sharing around mental healthcare among traditional healers in the general population in Kenya [17, 26].

Our results illustrate the need to train TBAs on the provision of evidence-based mental healthcare. If well trained and supervised, the TBAs could identify and address patient's psychosocial stressors and reduce prevalence of untreated mental health disorders among perinatal mothers. This will help reduce the work load at the primary healthcare facilities. Furthermore, this is helpful because the perinatal mothers are likely to visit and seek some sort of services from the TBAs and could benefit in terms of mental healthcare. Undeniably, training of the TBAs will have a multiplier effect on dissemination of messages on mental health and improve the community knowledge and practices in this regard. Collaboration and training of TBAs have been associated with significant positive change in knowledge and practices on antenatal healthcare [27].

## 5. Conclusion

This study identified that multiple stakeholders regard TBAs integration into mental health as a feasible step that may contribute to improved mental healthcare system. It was highlighted that TBAs have some experience which can be utilized in maternal mental healthcare. This would translate into early and improved disease detection, improved follow-up, and appropriate scaling up of mental health interventions. We recommend that the role of TBAs in community healthcare should be well defined. They should also be appreciated as important stakeholders in the healthcare structure, trained, and continuously supported in offering evidence-based mental health interventions to perinatal mothers in resource-poor settings.

## Figures and Tables

**Table 1:** Summary of interviews and FGDs from key stakeholders.

Study participants category	Data collection method	Number of groups or KIIs	No. of participants (number per group)
Traditional Birth attendants (TBAs)	FGD	6	59 (8-10)

Community Health Volunteers (CHVs)	FGD	3	29 (8-10)

Health Care Workers (HCWs)	FGD	3	21 (6-8)

Pregnant women	FGD	5	52 (6-13)

Postnatal women	FGD	6	44 (4-10)

Care givers (mothers/husbands) of pregnant and postnatal women	FGD	4	33 (8-10)

Opinion leaders, HCWs, husband, perinatal mother	KII	8	8(1)

Total number		35	246

FGDs: focus group discussions; KIIs: key informant interviews; TBAs: traditional birth attendants; CHVs: community health volunteers; HCWs: health care workers.

## Data Availability

The qualitative data used to support the findings of this study are available from the corresponding author upon request.
